# Automated interpretation of the pulmonary function test by a portable spirometer in Chinese adults

**DOI:** 10.1111/crj.13525

**Published:** 2022-07-22

**Authors:** Jun Zhou, Ping Wang, Leixin Guo, Jin Cao, Min Zhou, Ranran Dai

**Affiliations:** ^1^ Department of Respiratory and Critical Care Medicine, Ruijin Hospital, School of Medicine Shanghai Jiao Tong University Shanghai China

**Keywords:** automated interpretation, concordance, portable spirometer, pulmonary function test

## Abstract

**Introduction:**

A portable spirometer is a promising alternative to a traditional pulmonary function test (PFT) spirometer for respiratory function evaluation.

**Objectives:**

This study aimed to investigate the accuracy of automated interpretation of the PFT measured by a portable Yue Cloud spirometer in Chinese adults.

**Methods:**

The PFT was performed to evaluate subjects prospectively enrolled at Ruijin Hospital (*n* = 220). A Yue Cloud spirometer and a conventional Jaeger MasterScreen device were applied to each patient with a 20‐min quiescent period between each measurement. Pulmonary function parameters, including forced vital capacity (FVC), forced expiratory volume in the first second (FEV1), peak expiratory flow (PEF), maximal expiratory flow at 25%, 50%, and 75% of the FVC (MEF25, MEF50, and MEF75, respectively), and maximal mid‐expiratory flow (MMEF), were compared by correlation analyses and Bland–Altman methods. The Yue Cloud spirometer automatically interpreted the PFT results, and a conventional strategy was performed to interpret the PFT results obtained by the Jaeger machine. Concordance of the categorization of pulmonary dysfunction, small airway dysfunction, and severity was analyzed by the kappa (*κ*) statistic.

**Results:**

Significantly similar correlations of all variables measured with the two spirometers were observed (all *p* < 0.001). No significant bias was observed in any of the measured spirometer variables. A satisfactory concordance of pulmonary function and severity classification was observed between the automated interpretation results obtained with the Yue Cloud spirometer vs. a conventional spirometer interpretation strategy (all *κ* > 0.80).

**Conclusion:**

The portable Yue Cloud spirometer not only yields reliable measurements of pulmonary function but also can automatically interpret the PFT results.

## Graphical abstract

The accuracy of automated interpretation of pulmonary function test by the novel portable Yue Cloud spirometer was investigated in comparison with a conventional Jaeger MasterScreen device in Chinese adults. The results proved that the Yue Cloud spirometer not only yielded reliable measurements but also could automatically interpret the pulmonary function test results.

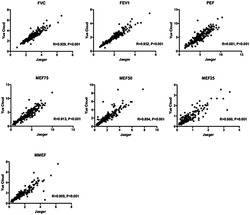

## 1 INTRODUCTION

Chronic obstructive pulmonary disease (COPD) is one of the leading causes of disability and death worldwide.^1^, ^2^ It is characterized by a progressive development and an irreversible airflow, causing 3 million deaths per year globally.^1^, ^3^ The prevalence of COPD in Chinese adults is estimated to be 8.6%, equating to 99.9 million people, according to a China Pulmonary Health study.^4^ However, the overall burden of COPD in China is underestimated due to a substantial underdiagnosis.^2^ It has been reported that only 9.7–12% of the spirometry‐detected COPD population had a prior pulmonary function test (PFT).^4^, ^5^ A PFT is routinely performed to evaluate the respiratory function in subjects with pulmonary disorders. However, the PFT is not widely applied, especially in rural China.^6^ The unavailability of traditional spirometers is partly due to the cost and complex nature of the operating system. In addition, the lack of well‐trained technicians and quality control limit the assessment of the PFT results.

A portable spirometer is a promising alternative to a traditional PFT spirometer. In Western countries, a portable spirometer has been proven to have a similar consistency compared with traditional spirometers.^7^, ^8^, ^9^ The Yue Cloud spirometer is a portable device made in China that can accurately and easily measure lung function.^10^ This easy‐to‐operate device is primarily indicated for use in screening and follow‐up of the population at risk of respiratory disease.^10^ However, scant evidence exists regarding the reliability of the automatic categorization of pulmonary dysfunction provided by a portable spirometer such as the Yue Cloud spirometer.

In the current prospective study, we aimed to validate the concordance of pulmonary dysfunction classification interpreted by an automated portable spirometer (Yue Cloud) compared with a traditional spirometer (Jaeger MasterScreen) in a Chinese cohort.

We present the following article in accordance with the STARD reporting checklist.

## 2 MATERIALS AND METHODS

### 2.1 Study design and patient population

This was a prospective, self‐control, single‐center study. We consecutively recruited adult subjects aged 18 and over who underwent a PFT at Shanghai Ruijin Hospital between February and July 2020. Those who failed to finish the PFT or without complete PFT results were excluded.

All included subjects (*n* = 220) received standardized instructions on the use of a spirometer. Two sets of PFTs were performed for each subject: the first by using a Jaeger MasterScreen machine (Serial No. 731267‐376400) and the second by using a Yue Cloud spirometer. The Yue Cloud spirometer is a small, handheld device consisting of a pressure sensor and a digital display, which has been proven to be a reliable alternative to the conventional device for the measurement of pulmonary function.^10^

The study was conducted according to the guidelines of the Declaration of Helsinki and approved by the Ethics Committee of Ruijin Hospital, Shanghai Jiao Tong University (approval number: 2020‐120). Informed consent was obtained from all participants.

### 2.2 Measurement

Spirometry variables, including the forced vital capacity (FVC), forced expiratory volume in the first second (FEV1), peak expiratory flow (PEF), maximal expiratory flow at 25%, 50%, and 75% of the FVC (MEF25, MEF50, and MEF75, respectively), and maximal mid‐expiratory flow (MMEF), were measured by the same technician on the same instruments at the pulmonary function laboratory. Small airway dysfunction was also measured. Measurement and calibration were strictly in compliance with the 2005 American Thoracic Society/European Respiratory Society guidelines. A quiescent period of 20 min was set before each measurement. The Yue Cloud spirometer automatically displayed the results of the measured parameters and the category of lung function impairment by the accompanying software. The measurements of pulmonary function obtained by the conventional Jaeger machine were evaluated by the technician based on the curve morphology.

### 2.3 Pulmonary dysfunction categorization

The pulmonary dysfunction was classified according to the Chinese guidelines of the PFT as follows^11^, ^12^: (1) obstructive impairment, FEV1/FVC < 92% of the predicted value, and (2) restrictive impairment, FEV1/FVC ≥ 92% of the predicted value and FVC < 80% of the predicted value for adults. Severity was classified as mild, moderate, moderate to severe, severe, or very severe, according to the FEV1 percentage of the predicted value: ≥70%, 60–69%, 50–59%, 35–49%, or <35%, respectively.

### 2.4 Quality control

The PFT results were categorized as Grade A, B, C, D, or F, according to the criteria for quality control based on the Chinese practice guidelines for PFT measurements (Table 1).^12^, ^13^

**TABLE 1:** Quality control criteria for the PFT measurement

Grade	Requirement
A	Reliable results (acceptable results obtained three times, two repeatable exhalations, best FEV1 and FVC within 0.150 L)
B	Reliable results (acceptable results obtained three times, two repeatable exhalations, best FEV1 and FVC results within 0.200 L)
C	At least two acceptable results obtained, best FEV1 and FVC results within 0.250 L
D	Unreliable results (at least two acceptable but unrepeatable results, or only one acceptable result)
F	Unreliable and unacceptable results

Abbreviations: FEV1, forced expiratory volume in the first second; FVC, forced vital capacity; PFT, pulmonary function test.

### 2.5 Statistical analysis

Continuous data were expressed as the mean ± standard deviation, whereas categorical data were expressed as a number with the percentage. Lung function parameters, including FVC, FEV1, PEF, MEF75, MEF50, MEF25, and MMEF, and small airway dysfunction were compared between groups. Pearson's correlation was used to analyze the relationships between variables. Meanwhile, the consistency of parameters measured by the Yue Cloud spirometer and the Jaeger device was assessed by Bland–Altman analysis.^14^ Bland–Altman plot is a graphical method to illustrate the agreement between two quantitative measurements. The graph is plotted on the *XY* axis, where *X* axis represents the difference of the two measurements and the *Y* axis shows the mean of the two measurements. The 95% limits of agreement (LoAs) are calculated to evaluate the differences between measurements by two methods. The concordance in diagnosis of lung function impairment between devices was assessed by Cohen's kappa statistic. The kappa coefficient (*κ*), indicating the strength of diagnosis agreement, was calculated. The kappa value was qualified on the basis of the magnitude as follows: 0.4–0.6, moderate agreement; 0.6–0.8, substantial agreement; and 0.8–1.0, almost perfect agreement.^15^ All statistical analyses were performed with SPSS 24.0 and GraphPad Prism 8.0. A *p* value <0.05 was considered statistically significant.

## 3 RESULTS

### 3.1 Demographic and clinical characteristics

The average age of all included subjects was 59 years old, with a median age of 63 (range: 18–85) years old. The male subjects accounted for 55.5% of the population. Among all 220 subjects, 159 (72.3%) had abnormal PFT results.

### 3.2 Spirometry measurements

**FIGURE 1:**
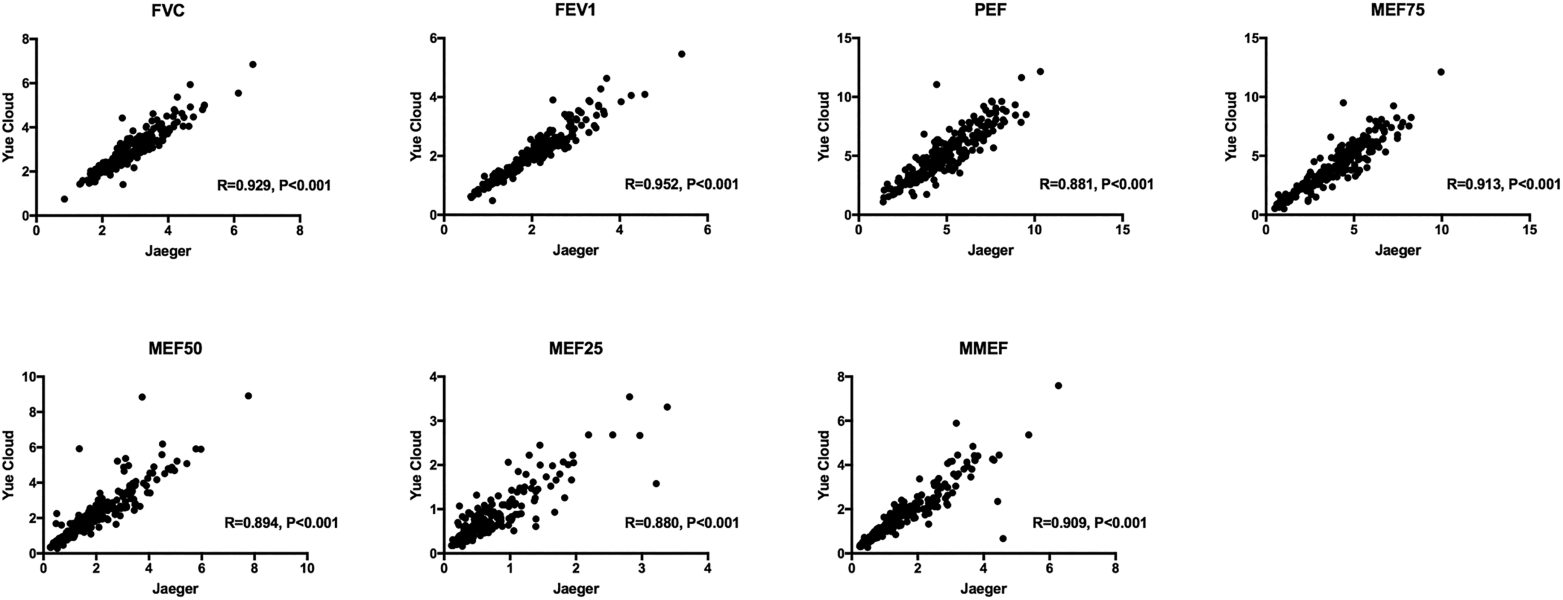
Correlation analysis of spirometry outcomes from the traditional Jaeger spirometer and the portable Yue Cloud spirometer. FEV1, forced expiratory volume in the first second; FVC, forced vital capacity; MEF, maximal expiratory flow at 25%, 50%, and 75% of the forced vital capacity (MEF25, MEF50, and MEF75, respectively); MMEF, maximal mid‐expiratory flow; PEF, peak expiratory flow

A high degree of agreement was detected in all spirometry outcomes (FVC, FEV1, PEF, MEF75, MEF50, MEF25, and MMEF) provided by the conventional Jaeger MasterScreen machine and the Yue Cloud spirometer. The correlations were all above 0.8, with a *p* value <0.001 (Figure 1).

**FIGURE 2:**
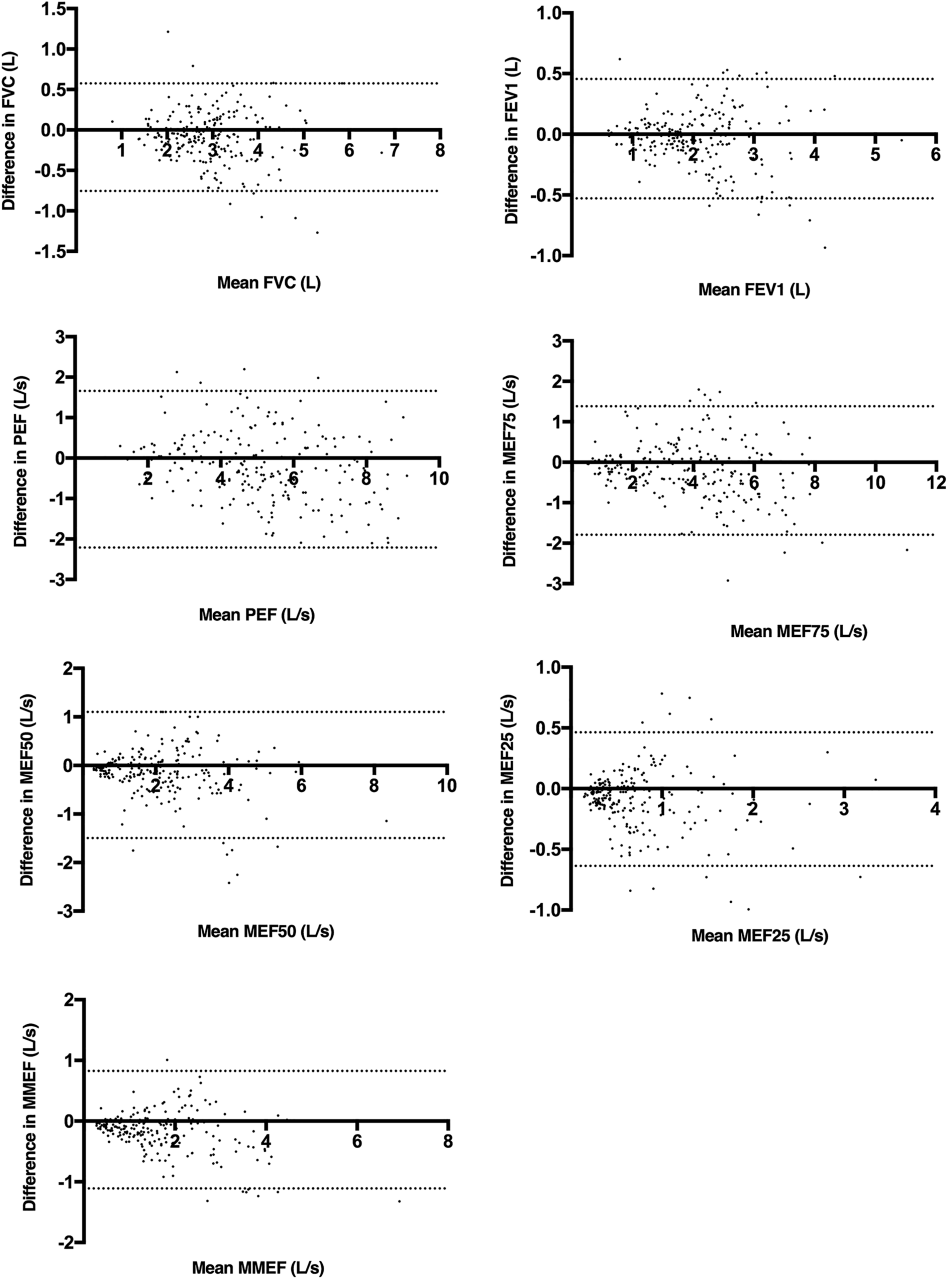
Bland–Altman plots with 95% limits of agreement summarizing the results of spirometry parameters measured by the traditional Jaeger spirometer and the portable Yue Cloud spirometer. The dotted lines represent the upper and lower LoAs. FEV1, forced expiratory volume in the first second; FVC, forced vital capacity; MEF, maximal expiratory flow at 25%, 50%, and 75% of the forced vital capacity (MEF25, MEF50, and MEF75, respectively); MMEF, maximal mid‐expiratory flow; PEF, peak expiratory flow

The Bland–Altman analyses showed that there was no significant bias in any of the spirometry measurements (FVC, FEV1, PEF, MEF75, MEF50, MEF25, and MMEF). Differences and 95% LoAs were displayed in Figure 2.

### 3.3 Concordance of pulmonary dysfunction and severity classification

The classifications of pulmonary dysfunction, small airway dysfunction, and severity, as measured by the Jaeger and Yue cloud spirometers, are displayed in Tables 2, 3, 4, respectively.

**TABLE 2:** Classification of pulmonary dysfunction

		Yue Cloud				Total
		Normal	Obstruction	Restriction	Mixed	
Jaeger	Normal	61 (27.7)	5 (2.3)	1 (0.5)	0 (0)	67 (30.5)
	Obstruction	0 (0)	52 (23.6)	0 (0)	1 (0.5)	53 (24.1)
	Restriction	5 (2.3)	2 (0.9)	42 (19.1)	1 (0.5)	50 (22.7)
	Mixed	1 (0.5)	1 (0.5)	1 (0.5)	47 (21.4)	50 (22.7)
Total		67 (30.5)	60 (27.3)	44 (20.0)	49 (22.3)	220 (100.0)

*Note*: Data are expressed as a number (%).

**TABLE 3:** Measurement of small airway dysfunction

		Yue Cloud		Total
		Without small airway dysfunction	Small airway dysfunction	
Jaeger	Without small airway dysfunction	101 (45.9)	4 (1.8)	105 (47.7)
	Small airway dysfunction	15 (6.8)	100 (45.5)	115 (52.3)
Total		116 (52.7)	104 (47.3)	220 (100.0)

*Note*: Data are expressed as a number (%).

**TABLE 4:** Classification of severity

		Yue Cloud					Total
		Mild	Moderate	Moderate to severe	Severe	Very severe	
Jaeger	Mild	66 (30.0)	5 (2.3)	0 (0)	0 (0)	0 (0)	71 (32.3)
	Moderate	1 (0.5)	19 (8.6)	1 (0.5)	1 (0.5)	1 (0.5)	23 (10.5)
	Moderate to severe	0 (0)	2 (0.9)	13 (5.9)	2 (0.9)	0 (0)	17 (7.7)
	Severe	0 (0)	0 (0)	1 (4.5)	26 (11.8)	0 (0)	27 (12.3)
	Very severe	0 (0)	0 (0)	0 (0)	0 (0)	10 (4.5)	10 (4.5)
Total		67 (30.5)	26 (11.8)	15 (6.8)	29 (13.2)	11 (5.0)	148 (67.3)

*Note*: Data are expressed as a number (%).

When measured by the Jaeger spirometer, the numbers of subjects diagnosed as having obstructive, restrictive, mixed pulmonary dysfunction, or normal pulmonary function were 53 (24.1%), 50 (22.7%), 50 (22.7%), and 67 (30.5%), respectively. The corresponding numbers of subjects measured by the Yue Cloud spirometer were 60 (27.3%), 44 (20.0%), 49 (22.3%), and 67 (30.5%), respectively (Table 2).

The Jaeger spirometer detected 115 (47.7%) subjects with small airway dysfunction, whereas 104 (47.3%) subjects were determined to have small airway dysfunction by the Yue Cloud spirometer. Among all these subjects, 100 (45.5%) were diagnosed with small airway dysfunction by both methods (Table 3).

Further severity classification revealed that 71 (32.3%), 23 (10.5%), 17 (7.7%), 27 (12.3%), and 10 (4.5%) subjects were categorized as having mild, moderate, moderate to severe, severe, and very severe disease, respectively, when measured by the Jaeger spirometer. The corresponding numbers of subjects were 67 (30.5%), 26 (11.8%), 15 (6.8%), 29 (13.2%), and 11 (5.0%), respectively, when measured by the Yue Cloud spirometer (Table 4).

Concordance verified by the Kappa statistic showed an excellent agreement in pulmonary dysfunction, small airway dysfunction, and severity evaluation between the Jaeger and Yue Cloud spirometers. The kappa values were all greater than 0.80 (pulmonary dysfunction, *κ* = 0.890; small airway dysfunction, *κ* = 0.828; and severity, *κ* = 0.874).

## 4 DISCUSSION

This prospective study determined the accuracy of pulmonary function assessment performed by a portable Yue Cloud spirometer and its diagnostic value of dysfunction categorization. Compared with the conventional Jaeger spirometer, the Yue Cloud spirometer performed well not only in the evaluation of lung function but also in the determination of classification. In terms of small airway dysfunction, severity classification, and routine dysfunction categorization, excellent concordance (*κ* > 0.8) was observed between both methods.

The population of this study included subjects with and without respiratory dysfunction. The proportion of patients with abnormal lung function accounted for more than 70% of the whole population. The balanced distribution of subjects ensures a comprehensive evaluation of the performance of the Yue Cloud spirometer. A previous study found a similar consistency of lung function variables, including FVC, FEV1, PEF, MEF75, MEF50, and MEF25, for adults.^10^ In this study, we further validated the good reliability of a portable spirometer on small airway dysfunction detection. A five‐grade classification of severity instead of a three‐grade classification also showed good agreement between the Yue Cloud spirometer and the Jaeger spirometer.

Computer‐aided diagnosis of pulmonary function has drawn increasing attention and is an advantage of the portable Yue Cloud spirometer. PFT interpretation requires expert knowledge, which is expensive and frequently unavailable at all levels of the healthcare system. The recent advances in machine learning and technology across the medical domain have stimulated a resurgence of artificial intelligence in PFT interpretation. The recently established machine learning framework to diagnose multiple obstructive lung diseases had a general accuracy of 68%, which outperformed the conventional interpretation strategy.^16^ The accuracy of pulmonary disease detection even reached up to 82% with the application of novel artificial intelligence‐based software.^17^ However, the current technology has yet to be sufficiently validated for real‐world clinical applications.^18^ In addition, most research on automated interpretation of PFT results has been performed in Western populations. In this study, in a general population of Chinese adults, we validated the good diagnostic value of the Yue Cloud spirometer. An excellent concordance, reflected by *κ* > 0.8, is a prerequisite of portable spirometry for future clinical applications.

The underdiagnosis of chronic pulmonary diseases such as COPD threatens public health. A large‐scale study investigating the prevalence of COPD has demonstrated that almost 100 million Chinese adults have COPD.^4^, ^5^ More importantly, the majority of this population is unaware of their condition.^5^ Only 9.7–12% of patients had undergone a PFT prior to the study.^4^, ^5^ The poorly developed healthcare system and the lack of experts limit the widespread use of PFT screening, especially in rural China. Therefore, the portable Yue Cloud spirometer will have broad application prospects. The automated interpretation of PFT results enables a prompt diagnosis of pulmonary diseases, without the need for professional personnel.

This study had several limitations. First, the Yue Cloud spirometer cannot measure the diffusion function. Second, we only validated the diagnostic accuracy of the Yue Cloud spirometer in adults. Its performance for pediatric patients was not analyzed. Third, this was a single‐center study. Large‐scale validation studies should be conducted in the future.

## 5 CONCLUSIONS

Satisfactory accuracy of pulmonary function classification was automatically achieved by the portable Yue Cloud spirometer. The portable Yue Cloud spirometer is an ideal alternative to the conventional device for the measurement and classification of pulmonary function.

## CONFLICTS OF INTEREST

The authors declare that they have no conflicts of interest.

## ETHICS STATEMENT

The study was conducted according to the guidelines of the Declaration of Helsinki and approved by the Ethics Committee of Ruijin Hospital, Shanghai Jiao Tong University (approval number: 2020‐120).

## PATIENT CONSENT STATEMENT

Informed consent was obtained from all participants.

## AUTHOR CONTRIBUTIONS

*Conception and design*: R. D. and M. Z. *Administrative support*: P. W. *Provision of study materials or patients*: P. W. and J. C. *Collection and assembly of data*: L. G. *Data analysis and interpretation*: J. Z. and R. D. *Manuscript writing*: all authors. *Final approval of manuscript*: all authors.

## Data Availability

The datasets generated and analyzed during the current study are available from the corresponding author on reasonable request.
